# Long-term survival of elderly patients after intensive care unit admission for acute respiratory infection: a population-based, propensity score-matched cohort study

**DOI:** 10.1186/s13054-020-03100-4

**Published:** 2020-06-29

**Authors:** Antoine Guillon, Coralie Hermetet, Kimberly A. Barker, Youenn Jouan, Christophe Gaborit, Stephan Ehrmann, Yannick Le Manach, Pierre-François Dequin, Leslie Grammatico-Guillon

**Affiliations:** 1 CHRU de Tours, Service de Médecine Intensive Réanimation, INSERM U1100, Centre d’Etude des Pathologies Respiratoires, CRICS-TriggerSEP Research Network, Université de Tours, 2 Bd Tonnellé, F-37044 Tours Cedex 9, France; 2 CHRU de Tours, Epidémiologie des Données Cliniques (EpiDcliC), Service d’Information Médicale, d’Épidémiologie et d’Économie de la Santé, Université de Tours, Tours, France; 3 EA EES, Université de Tours, Tours, France; 4 Pulmonary Center, Boston University School of Medicine, Boston, MA USA; 5 Department of Microbiology, Boston University School of Medicine, Boston, MA USA; 6 CIC INSERM 1415, Tours, France; 7 Departments of Anesthesia & Clinical Epidemiology and Biostatistics, Faculty of Health Sciences, Michael DeGroote School of Medicine, McMaster University and the Perioperative Research Group, Population Health Research Institute, Hamilton, ON Canada

**Keywords:** Respiratory infection, Intensive care unit, Elderly, Epidemiology

## Abstract

**Background:**

Intensive care unit (ICU) hospitalisations of elderly patients with acute respiratory infection have increased, yet the long-term effects of ICU admission among elderly individuals remain unknown. We examined differences over the 2 years after discharge in mortality, healthcare utilisation and frailty score between elderly survivors of ARI in the ICU and an elderly control population.

**Methods:**

We used 2009–2017 data from 39 hospital discharge databases. Patients ≥ 80 years old discharged alive from ICU hospitalisation for acute respiratory infection were propensity score-matched with controls (cataract surgery) discharged from the hospital at the same time and adjusted for age, sex and comorbidities present before hospitalisation. We reported 2-year mortality and compared healthcare utilisation and frailty scores in the 2-year periods before and after ICU hospitalisation.

**Results:**

One thousand two hundred and twenty elderly survivors of acute respiratory infection in the ICU were discharged, and 988 were successfully matched with controls. After discharge, patients had a 10.1-fold [95% CI, 6.1–17.3] higher risk of death at 6 months and 3.6-fold [95% CI, 2.9–4.6] higher risk of death at 2 years compared with controls. They also had a 2-fold increase in both healthcare utilisation and frailty score in the 2 years after hospital discharge, whereas healthcare utilisation and frailty scores among controls were stable before and after hospitalisation.

**Conclusions:**

We observed a substantially increased rate of death in the years following ICU hospitalisation for elderly patients along with elevated healthcare resource use and accelerated age-associated decline as assessed by frailty score. These findings provide data for better informed goals-of-care discussions and may help target post-ICU discharge services.

## Introduction

The ageing of the population is a global trend of critical importance, with the number of individuals aged 80 years or older increasing the most rapidly (3.8% increase/year) [1]. This worldwide ageing has major consequences on health systems including increasing elderly patient admissions in hospitals and intensive care units (ICUs) [2, 3]. ICU admission of elderly patients is controversial, but randomised controlled studies are difficult to design due to ethical limitations [4]. Indeed, conflicting results are present in the literature: some studies report that advanced age is itself a risk factor for ICU mortality and poor long-term quality of life due to persistent functional impairments [5–8], while other studies argue that chronological age alone should not be considered the sole criterion to preclude ICU admission [9, 10].

Most studies focusing on post-ICU effects among the elderly have considered elderly patients as a single entity. However, we feel it is questionable to discuss hospital admission policies for patients based solely on their advanced age without considering the acute disease leading to admission. Discussions of these topics should be disease-specific, and a focus on respiratory infections is of particular interest because of the strong association of these diseases with old age [11]. Consequently, we previously demonstrated a substantial increase in hospitalisations for acute respiratory infection (ARI) over 10 years (2006–2015), with a change in the ICU admission policy for elderly patients leading to an important rise in ICU resource utilisation [11]. For example, the number of nonagenarians hospitalised in ICU for ARI was 5.8-fold higher in 2015 than in 10 years earlier. However, the long-term effects of ICU admission for elderly patients with ARI have never been demonstrated.

Assessing the burden of post-ICU effects among elderly patients is particularly difficult due to the impossibility of comparing ICU-treated individuals with the most appropriate control group. Comparing elderly patients hospitalised for ARI according to whether or not they were admitted to the ICU is importantly biassed: non-ICU patients include patients that did not meet the severity criteria for ICU admission along with other cases that are sometimes severe but received only palliative care. Comparing ICU-treated patients from different age groups also does not provide useful information because of the increased risks of death and functional impairment that clearly accompany increased age.

The objective of this study was to examine the 2-year outcomes among elderly patients discharged from the hospital after being admitted to the ICU for ARI. To describe the gap in morbi-mortality between the ICU survivors and the theoretical medical condition expected at this age, we studied differences between elderly survivors of ARI and a control population representative of the physiologic effects of ageing, and matched on age, sex and preexisting comorbidities. The primary outcome was mortality, and the secondary outcomes were healthcare utilisation and frailty score over the 2 years after discharge.

## Methods

### Data source

A population-based cohort study was performed from January 1, 2009, to December 31, 2017, in the Centre-Val de Loire region of France (2.5 million inhabitants), which is served by 39 hospitals. We used data collected from the PMSI (*Programme de Médicalisation des Systèmes d’Information*) national hospital discharge database. This national database is made based on the mandatory notification of each hospital stay for all French public or private hospitals. All hospitalisation information is stored in a coded summary using the International Classification of the Diseases, tenth revision (ICD-10) and the French current procedural terminology for what occurred during the hospital stay. All patients are assigned a unique identification number, allowing the same individual to be followed over time [12].

### Case definitions

We defined cases of “ARI” and “cataract surgery” using an ICD-10 algorithm based on the coding resume and the French current procedure terminology coded featuring the discharge summary. ARI included diagnosis codes for acute exacerbation of chronic obstructive pulmonary disease (AECOPD) and community-acquired pneumonia (CAP) (ICD-10 diagnosis codes provided in Additional file 1). Hospitalised patients who received at least one of these ICD-10 diagnosis codes as (1) the primary diagnosis in their discharge summary or (2) the secondary diagnosis with a primary diagnosis of respiratory failure were defined as having been hospitalised with ARI. The selection of hospital stays for “cataract surgery” was performed based on the codes for cataract surgery in discharge summaries. For each patient, the following data were extracted: age, sex, primary diagnosis, comorbidities (see Additional file 1 for ICD-10 diagnosis codes used) and hospital frailty risk score at admission (according to the ICD-10 codes from the two previous years of hospital discharge summaries and measured as a continuous quantitative score from 0 to 99 [13]). The procedures performed during the ICU stay and the hospitalisation characteristics were also recorded for the ARI patients.

### Study population

We included all patients aged 80 years or older who were hospitalised at least one time between January 1, 2011, and December 31, 2013, and had one of two clinical profiles: (1) patients admitted to the ICU for ARI and discharged home alive as the population of interest and (2) patients admitted for cataract surgery and discharged home as the control population to address our expectation of spontaneous complications and death attributable to the age of this population; we matched the sex, age and comorbidities of the ARI patients with this control population selected from patients undergoing cataract surgery at the same time. We made the assumption that cataract surgery had an insignificant impact on mortality [13] and assumed that these control patients were an accurate representation of the physiological age-related prevalence of chronic diseases and mortality.

For each patient hospitalised with ARI in the ICU and discharged alive from the hospital, a corresponding comparison patient hospitalised for cataract surgery (1:1 ratio) was selected on the basis of the nearest propensity score using the one-to-one nearest neighbour method (with a calliper of 0.002 of the standard deviation of the propensity score on the logit scale [14]) with no replacement. Matching was performed based on preexisting conditions identified over a 2-year period prior to the hospitalisation event (age, sex, frailty score, chronic heart diseases, chronic lung diseases and cancers). Hence, we used the long-term outcomes (mortality, healthcare utilisation, frailty score evolution) as proxy measures of the burden of post-ICU effects for patients 80 years of age or older who were hospitalised for ARI. After matching, the balance of covariates between the two groups was assessed using the standardised differences expressed as a percentage overall and for each covariate using the L1 measure (for which a value of 0 indicates identical distributions between groups and 1 indicates complete imbalance) and the post-matching C-statistic (for which a standardised mean difference of 0.05 or less indicates a negligible difference between the means of the two groups and a perfect balance).

Data on the propensity-matched patients with “ARI” or “Cataract” patients were then collected for the 2-year period preceding the hospitalisation (2009–2010) and for the 2-year period following hospital discharge (2014–2015). Mortality was studied over a 2-year period (2014–2015). To accurately achieve this aim, the ICD-10 algorithm examined the 4-year period (2014–2017) to capture more information on the living or dead status (i.e.*,* being alive in 2017 indicated an individual was alive at the end of 2015).

### Outcomes

We furthermore studied the 2-year risk of mortality for patients 80 years or older who were discharged home alive after ICU stay for ARI compared with the expected mortality risk of this age group as determined from the controls. Mortality refers to mortality at hospital, which was defined as death during readmission during the follow-up period.

We studied healthcare utilisation in the 2-year periods preceding and following the hospital stay of interest in the matched population. A patient’s care utilisation was determined by the number of days spent as an inpatient during these periods (i.e., the percentage of inpatient days per quarter). Inpatient care included outpatient visits (each one counting for 1 day of healthcare use), ambulatory care at a hospital (1 day), hospital stays (number of days spent at a hospital) and stays at rehabilitation facilities (number of days spent in the facility). Each patient acted as their own control between the two periods. We also assessed the evolution of the frailty score from the 2-year period before the hospital stay of interest through the 2-year period after this hospital stay.

### Ethical approval

No nominative, sensitive or personal data on patients have been collected. Our study involves the reuse of already recorded and anonymised data. The study falls within the scope of the French Reference Methodology MR-005 according to 2016–41 law dated 26 January 2016 on the modernisation of the French health system, which require neither information nor non-opposition of the included individuals. Access to linked anonymous file in the PMSI databases was approved by the French National Commission for Data Protection and Liberties (CNIL MR-005 number 4116221019).

### Statistical analyses

Continuous variables were compared using parametric or nonparametric methods, as appropriate, for paired (paired Student *t* tests or Wilcoxon matched-pairs signed rank tests) or unpaired (Student *t* tests or Mann-Whitney tests) data. Qualitative variables were also compared using parametric or nonparametric methods, as appropriate, for paired (McNemar tests or Fisher tests) or unpaired (*X*^2^ tests or Fisher tests) data. Kaplan-Meier curves were used to visualise the differences in survival between the ARI-ICU and control populations with log rank estimates; whether a patient was lost to follow-up, died or survived was specified every 6 months. Hazard ratios (HRs) and the 95% CIs for primary outcomes were computed using Cox modelling of the matched population. *P* values were 2-tailed, and values less than .05 were considered significant. Relative risks of death between the two matched groups were assessed at 1, 2 and 4 years after hospitalisation. Statistical analyses were carried out using R software [15] version 3.1, and matching procedures were performed using the MatchIt package [16], version 2.4-21. Hazard ratios were determined using the survival package [17, 18].

## Results

### Matched study population

During the inclusion period (2011–2013), 12,646 patients 80 years or older were hospitalised for ARI. Among them, 1658 patients were hospitalised in ICU and 438 (26.4%) of those patients died during the stay. Table 1 reports the baseline characteristics and specific care support provided in ICU for the 1220 patients with ARI who were discharged alive from the hospital. During the same period, 18,921 patients were admitted for cataract surgery. The mortality during hospitalisation for cataract surgery was 0.02% (3 patients). Using the 1220 patients discharged alive from ICU hospitalisation for ARI, the matching procedure resulted in a propensity-matched cohort of 1976 patients of 80 years or older, including 988 patients hospitalised for ARI in ICU and 988 patients who underwent cataract surgery. After matching on preexisting conditions identified before the hospitalisation, there was no statistically significant difference in baseline characteristics between the patient groups (Table 2). The initial ARI cohort and the matched ARI patients are presented in Table 1.

**Table 1:** Baseline characteristics of elderly patients with ARI who were discharged from the hospital ICU. Patients’ characteristics and specific care supports during the ICU stay are presented for both the total ARI cohort and the ARI cohort after matching was applied

	ARI patients (***n*** = 1220)	ARI patients after matching (***n*** = 988)
**Sex ratio (M/F)**	1.2	1.0
**Comorbidities (*n*, %)**		
Chronic pulmonary diseases	274 (22.5)	109 (11.0)
Diabetes	181 (14.8)	124 (12.5)
Obesity	122 (10.0)	74 (7.5)
Chronic liver diseases	14 (1.2)	11 (1.1)
Cancer	140 (11.5)	91 (9.2)
Chronic renal diseases	142 (11.6)	75 (7.6)
Chronic heart diseases	530 (43.4)	336 (34.0)
Neurological diseases	212 (17.4)	126 (12.8)
**SAPS II (mean [95% CI])**	38.2 [37.5; 39.0]	38.3 [37.5; 39.1]
**Specific care supports**		
Mechanical ventilation		
Invasive (*n*, %); duration (day, mean [95% CI])	194 (15.9); 6.5 [5.6; 7.4]	161 (16.3); 6.6 [5.6; 7.6]
Non-invasive (*n*, %); duration (day, mean [95% CI])	355 (29.1); 3.6 [3.2; 3.9]	269 (27.2); 3.2 [2.9; 3.6]
Vasopressor (*n*, %)	127 (10.4)	108 (10.9)
Renal replacement therapy (*n*, %)	24 (2)	17 (1.7)
**Length of stay (day)**		
In all inpatient units: mean [95% CI]	16.4 [15.7; 17.1]	16.3 [15.6; 17.1]
In ICU: mean [95% CI]	6.3 [5.9; 6.7]	6.3 [5.9; 6.7]

**Table 2:** Pre-hospitalisation patient characteristics of the matched study population. Patients discharged home after ICU hospitalisation for ARI or cataract surgery (control patients) during the same period were selected. The matching was performed based on preexisting conditions identified during the 2-year period before the hospitalisation

	Patients with ARI	Patients with cataract surgery	***p*** value
**Number**	988	988	
**Age (years, mean [95% CI])**	85.1 [84.8; 85.3]	85.2 [84.9; 85.4]	0.623
**Female sex (n, %)**	494 (50.0)	495 (50.1)	1.000
**Comorbidities (n, %)**			
Chronic heart failure	336 (34.0)	356 (36.0)	0.370
Chronic pulmonary diseases	109 (11.0)	105 (10.6)	0.828
Cancer	91 (9.2)	79 (8.0)	0.378
**Frailty score (mean [95% CI])**	2.88 [2.62; 3.15]	2.78 [2.50; 3.06]	0.608

### Long-term mortality

**Fig. 1:**
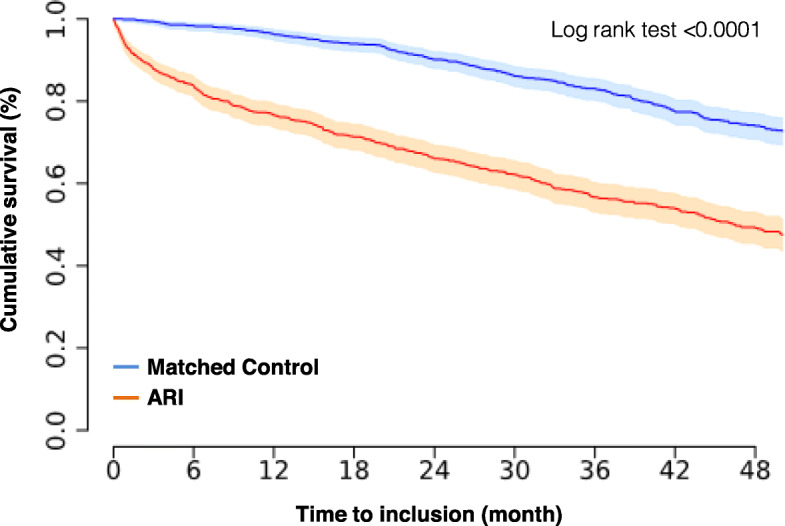
Kaplan-Meier curves showing the cumulative probabilities of survival. Light colours mark the 95% CIs of the corresponding curves (ARI, acute respiratory infection)

The Kaplan-Meier curves for elderly patients discharged alive after ICU hospitalisation for ARI and for the control population showed a significant difference in mortality for these two groups (log rank test < 0.0001, Fig. 1). Patients discharged alive after ARI in the ICU had a 10.1-fold [95% CI, 6.1–17.3] higher risk of death at 6 months (mortality rates were 1.7% (15/897) and 17.2% (145/844) in matched controls and ARI patients, respectively) and a 3.6-fold [95% CI, 2.9–4.6] higher risk of death at 2 years (mortality rates were 10.3% (85/822) and 37.5% (283/754) in matched controls and ARI patients, respectively).

### Healthcare utilisation

**Fig. 2:**
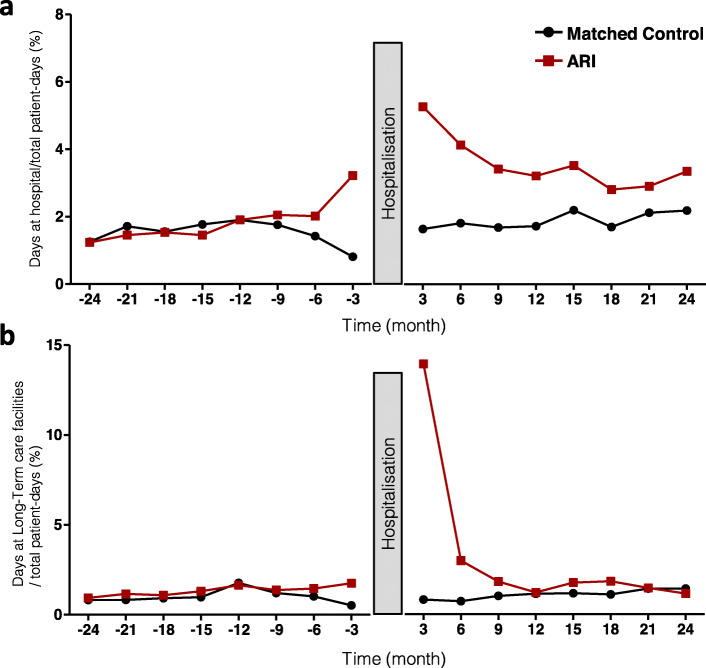
Healthcare utilisation before and after initial hospital stay. The number of days spent as an inpatient in a hospital (**a**) or in long-term care facilities (**b**) during the 2-year periods before and after the initial stay (i.e., the percentage of days spent in the hospital per quarter)

Figure 2a reports the healthcare utilisation during the 2-year periods before and after the initial hospital stay. The healthcare utilisation after ARI substantially increased in the 6 months after discharge and remained higher than the utilisation in the matched control population during the 2-year follow-up period (*P* < 0.0001). Comparatively, for the matched controls, the mean percentage of days spent in the hospital during the 2-year period after surgery slightly increased after the cataract surgery, but this increase did not reach statistical significance (1.53 ± 0.35% of days before the procedure vs 1.88 ± 0.24% of days after the procedure, *p* = 0.07). In the patients with ARI, we demonstrated a 2-fold increase in healthcare utilisation after being discharged alive from the hospital: the mean percentage of days spent in the hospital per quarter was 1.86 ± 0.63% of days before the hospitalisation and 3.58 ± 0.79% of days after the hospitalisation over a 2-year period (*p* = 0.005). The number of days spent in a long-term care hospital drastically increased during the first 6 months post-discharge from ICU hospitalisation for ARI and then returned to the control mean estimates (estimates for the matched controls and for ARI patients during the pre-hospitalisation comparative period) (Fig. 2b).

### Frailty risk score

**Fig. 3:**
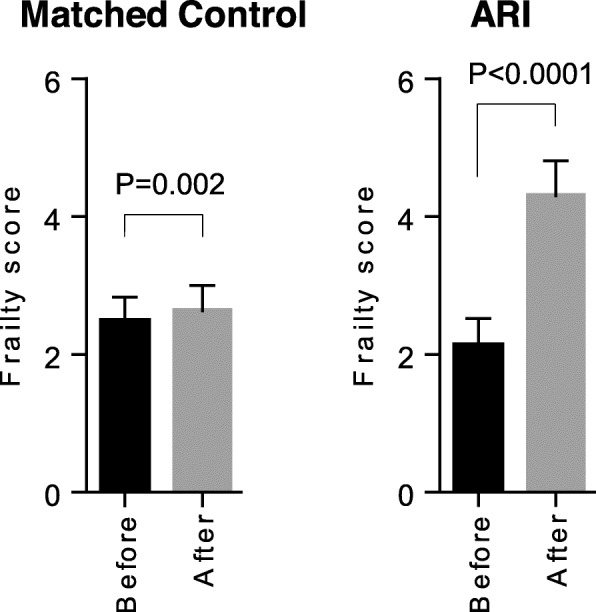
Evolution of the frailty score during the 2-year periods before and after the initial hospital stay. Paired analyses performed only in patients still living 2 years after being discharged from the hospital (*n* = 737 for matched controls, *n* = 472 for ARI)

Since frailty is potentially a determinant of the care resources needed, the evolution of hospital frailty risk score is presented in Fig. 3. We observed a 2-fold increase in the hospital frailty risk score among the ARI patients in the 2 years post-ICU, from 2.17 [95% CI, 1.83–2.52] to 4.28 [95% CI, 3.76–4.81] (paired analysis of 472 patients still living 2 years after being discharged from the hospital, *p* < 0.0001); whereas the hospital frailty score of the controls slightly increased in the 2 years post-hospitalisation + 4% (from 2.52 [95% CI, 2.22–2.83] to 2.62 [95% CI, 2.23–3.00] (paired analysis of 737 patients still living 2 years after cataract surgery, *p* = 0.002)).

## Discussion

We demonstrated that patients over 80 years old hospitalised in ICU for ARI were discharged alive from the hospital in 75% of cases. However, these patients had a 10-fold increased risk of death 6 months post-hospitalisation and 3.6-fold increased risk of death at 2 years post-hospitalisation as compared to a control population. Further, during the 2-year post-ICU period, healthcare utilisation and frailty score doubled among the ARI patient group.

We performed a propensity-matched population analysis of patients with ARI admitted in ICU compared to patients undergoing cataract surgery, which was assumed to have no effect on survival or future re-hospitalisation. The insignificant mortality observed during the stays for cataract surgery combined with the comparable pre- and post-healthcare utilisation and frailty score evaluations confirmed this population as a relevant control. Importantly, matching was performed based on comorbidities that were present before the critical illness of ARI patients. As the populations were comparable in terms of age, sex and underlying diseases before the acute illness, one can attribute the excess mortality observed after hospital discharge to the severe illness and associated treatment (ARI requiring ICU hospitalisation) and not to spontaneous ageing or consequences of preexisting underlying conditions. Our study shows that an elderly patient being discharged alive from ICU is clearly not the end of the story, but rather an important turning point in life. The main findings of this study could be expressed from binary viewpoints. On one hand, as a group, a substantial proportion of elderly patients will die in the 6 months after discharge from the ICU. Moreover, the survivors will endure a critical decrease in their quality of life, as suggested by the worsening of the frailty score and the increase in healthcare use we observed after hospitalisation. On the other hand, on an individual level, many elderly patients surviving ICU hospitalisation will live more than 4 years after discharge (239 patients among 988). These findings highlight the need for early consideration of the management of frailty and newly developed comorbidities after ICU discharge, and more generally for management of the overall post-ICU rehabilitation process. Taking the management of hip fractures in elderly patients as an example, close cooperation between emergency physicians, anaesthesiologists, intensivists, surgeons, geriatricians and rehabilitation physicians has proven to drastically reduce the risk of death in the months after hospital discharge [19, 20]. ARI is a severe and frequent disease among the elderly and requires the same attention. Another finding of our study is that 6 months is a more appropriate time frame to define the true ICU mortality for an elderly population, rather than the usual 30-day mortality [19, 21].

The present study has several strengths. First, we defined an inventive control population and matched based on preexisting conditions present before the occurrence of critical illness. By using medico-administrative databases, we were able to compare pre- and post-hospitalisation trajectories and healthcare utilisation. Previous cohort studies have provided substantial knowledge on the long-term outcomes after hospital discharge [22–24]; however, prospective cohort studies are limited in the amount of information they can provide from long-term follow-up because of the lack of prehospital trajectory data [25]. Moreover, many of these long-term follow-up studies lack appropriate control groups for comparison. Consequently, there are uncertainties regarding the extent to which the morbidity and mortality observed after hospital discharge in these studies can be attributed to the critical illness and the extent to which preexisting underlying conditions contributed to such findings (which is a major limitation for elderly populations with expected comorbidities and a high mortality rate). Second, our study used a large contemporary database of 39 acute healthcare facilities in which collection of data on every hospital stay is mandatory. These data are generated based on the patient’s routine care without any intervention affecting the usual methods of patient care. This study design could be assimilated as a real-life study, which contributes to the high generalisability of the results [26]. Third, in addition to survival analyses, this study assessed healthcare utilisation and frailty score evolution, which are important and relevant outcomes in an elderly population. The frailty score measures susceptibility due to the age-associated decline in reserve and function in a wide range of physiological systems [27]. Frailty was associated with an increased risk of mortality in critically ill patients older than 80 years [28] and generally in elderly patients [29]. The doubling of the frailty score that we observed in the ICU survivors is consistent with the doubling of healthcare utilisation observed over the same time frame.

This study also has several limitations. First, the use of administrative hospital databases introduced an inherent bias that should be taken into consideration. The strengths and limitations of using healthcare databases for epidemiological purposes have already been extensively discussed [30–34]. Second, the mortality was estimated based on readmissions and mortality at hospital. Out-of-hospital deaths were not recorded, and we were thus unable to calculate the actual mortality. Based on data from the “Fin de vie en France” [End of life in France] survey, we know that dying people in France do not stay in their own homes and mostly end up in a hospital: only 14% remains at home throughout the last month of life [35]. Additionally, we expect that the rate of out-hospital deaths was similar in our experimental and control groups. Indeed, our algorithm captured the vast majority of the deaths, and we can assume that the observed increased risk of death is accurate. Third, we cannot completely exclude the possibility that there were factors affecting only one group (ARI or cataract group) that were not measured in the data available for matching. The use of two approaches (matching and pre/post analysis) demonstrated consistency and was against this latest hypothesis. However, it should be noted that only the main comorbidities, as opposed to all comorbidities, were used in matching (Table 2). The matching process also may have excluded patients with more severe illness (see Table 1). This may have led to an underestimation of the gap between morbi-mortality of elderly survivors of ARI in the ICU and theoretical values expected for this age group (represented by the control cohort). Moreover, patients were not matched on socioeconomic status and residential status (assisted living, etc.), both of which would have at least some impact on the dependency measures. Fourth, it is likely that triage based on the appropriateness of ICU admission for elderly patients was very selective. Elderly individuals are admitted to ICU only if a high likelihood of survival is expected a priori. Consequently, our results can only be extrapolated to health systems with equivalent elderly triage processes. In particular, this study is specific to the French healthcare setting and may not be generalisable. Finally, we did not include elderly patients with ARI who were admitted in the general ward as controls. Having an intermediate group of patients who survived ARI without ICU admission would have conferred additional strength to this study and given insight to any potential “dose-response” where higher illness severity potentially increases the long-term burden on elderly patients recovered from acute illness. However, patients were highly heterogeneous in the general ward and were comprised of those (i) not severe enough for ICU admission and (ii) meeting the criteria for ICU admission but who were too old and/or had too much comorbidity. The survivors in this heterogeneous population are difficult to characterise, because their reasons for ICU non-admission cannot be captured by the ICD-10 algorithms. Moreover, these two populations (“elderly in general ward” and “elderly in ICU”) were by definition different, because the physicians in charge of each patient initially made a call as to whether ICU admission was appropriate. Thus, trying to match these two groups by a propensity score is likely inappropriate. A prospective randomised controlled trial is probably the ideal method to answer the question of ICU benefits and the burden of post-ICU effects, but ethical concerns limit the feasibility of this approach.

## Conclusion

Our data provide insight into outcomes during a 2-year follow-up of patients over 80 years old who were discharged alive from ICU after severe respiratory infection. We demonstrated an important increase in the rate of death in the years after hospital discharge along with elevated healthcare resource use and accelerated age-associated decline as assessed by the frailty score. These findings provide data for more informed goals-of-care discussions and may help target post-ICU discharge services for these high-risk patients.

## Supplementary information

**Additional file 1: Supplementary information on Methods.** ICD-10 diagnosis codes used.

## Data Availability

The data that support the findings of this study are available from CVL Hospitals, but restrictions apply to the availability of these data and so are not publicly available. However, data are available from the authors upon reasonable request and with the permission of the institution.

## Abbreviations

ARI: Acute respiratory infection
CNIL: Commission Nationale de l’Informatique et des Libertés
CVL: Centre Val-de-Loire
HDD: Hospital discharge database
ICU: Intensive care unit
INSEE: Institut National de la Statistique et des Etudes Economiques
PMSI: Programme de Médicalisation des Systèmes d’Information
SD: Standard deviation
