# Supplementary material for: Long-term survival of elderly patients after intensive care unit admission for acute respiratory infection: a population-based, propensity score-matched cohort study
Source: Crit Care. 2020 Jun 29;24:384. doi: 10.1186/s13054-020-03100-4 (PMC7325055; doi:10.1186/s13054-020-03100-4)
Supplement: Supplementary file 1 — Additional file 1: Supplementary information on Methods. ICD-10 diagnosis codes used. [file 13054_2020_3100_MOESM1_ESM.docx]

**Long-term survival of elderly patients after intensive care unit admission for acute respiratory infection: a population-based, propensity-score matched cohort study.** A. Guillon, C. Hermetet, K. A. Barker, Y. Jouan, C. Gaborit, S. Ehrmann, Y. Le Manach, P.F. Dequin, L. Grammatico-Guillon

**ONLINE SUPPLEMENT.**

**METHODS**

**Case definitions**

We defined cases of « ARI » and « cataract surgery » using an ICD-10 algorithm based on the coding resume and the French current procedure terminology coded featuring the discharge summary. ARI included diagnosis codes for acute exacerbation of chronic obstructive pulmonary disease (AECOPD) and community-acquired pneumonia (CAP). The codes recorded were: AECOPD (J440, J441), CAP (bacterial CAP: J13, J14, J150–J160, J170; viral CAP: J09, J100, J101, J108, J110, J111, J118, J120–J123, J128, J129; unspecified aetiology of CAP: J168, J172, J173, J178, J180–J182, J188, J189, J690, J851). Hospitalised patients who received at least one of these ICD-10 diagnosis codes as (1) the primary diagnosis in their discharge summary or (2) the secondary diagnosis with a primary diagnosis of respiratory failure (ICD-10 code J960) were defined as having been hospitalised with ARI. The selection of hospital stays for « cataract surgery » was performed based on the codes for cataract surgery in discharge summaries (02C05).

# Definition of comorbidities.

# A - CARDIOVASCULAR :

### 1 - ISCHEMIC HEART DISEASE

#### I20.x - Angina pectoris

#### I25.x - Chronic ischemic heart disease

#### Z95.5 - Presence of coronary angioplasty implant and graft

### 2 - CARDIAC, ARRHYTHMIA

#### I44.1 - Atrioventricular block, second degree

#### I44.2 - Atrioventricular block, complete

#### I44.3 - Other and unspecified atrioventricular block

#### I45.6 - Pre-excitation syndrome

#### I45.9 - Conduction disorder, unspecified

#### I47.x - Paroxysmal tachycardia

#### I49.x - Other cardiac arrhythmias

#### R00.0 - Tachycardia, unspecified

#### R00.1 - Bradycardia, unspecified

#### R00.8 - Other and unspecified abnormalities of heart beat

#### Z95.0 - Tachycardia, unspecified

### 3 - ATRIAL FIBRILLATION

#### I48.x - Atrial fibrillation and flutter

### 4 - CHRONIC HEART FAILURE

#### I11.0 - Hypertensive heart disease with (congestive) heart failure

#### I13.0 - Hypertensive heart and renal disease with (congestive) heart failure

#### I13.2 - Hypertensive heart and renal disease with both (congestive) heart failure and renal failure

#### I42.0 - Dilated cardiomyopathy

#### I42.5 - Other restrictive cardiomyopathy, Constrictive cardiomyopathy.

#### I42.6 - Alcoholic cardiomyopathy

#### I42.7 - Cardiomyopathy due to drugs and other external agents

#### I42.8 - Other cardiomyopathies

#### I42.9 - Cardiomyopathy, unspecified; Cardiomyopathy.

#### I43.x - Cardiomyopathy in diseases classified elsewhere

### 5 - VALVULAR DISEASE

#### I05.x - Rheumatic mitral valve diseases

#### I06.x - Rheumatic aortic valve diseases

#### I07.x - Rheumatic tricuspid valve diseases

#### I08.x - Multiple valve diseases

#### I34.x - Nonrheumatic mitral valve disorders

#### I35.x - Nonrheumatic aortic valve disorders

#### I36.x - Nonrheumatic tricuspid valve disorders

#### I37.x - Pulmonary valve disorders

#### I38.x - Endocarditis, valve unspecified

#### I39.x - Endocarditis and heart valve disorders in diseases classified elsewhere

#### Q23.0 - Congenital stenosis of aortic valve

#### Q23.1 - Congenital insufficiency of aortic valve

#### Q23.2 - Congenital mitral stenosis

#### Q23.3 - Congenital mitral insufficiency

#### Z95.2 - Presence of prosthetic heart valve

#### Z95.3 - Presence of xenogenic heart valve

#### Z95.4 - Presence of other heart-valve replacement

### 6 - PERIPHERAL VASCULAR DISEASE

#### I70.x - Atherosclerosis

#### I70.1 - Thoracic aortic aneurysm, without mention of rupture

#### I70.4 - Abdominal aortic aneurysm, without mention of rupture

#### I70.6 - Thoracoabdominal aortic aneurysm, without mention of rupture

#### I70.9 - Aortic aneurysm of unspecified site, without mention of rupture

#### I73.1 - Thromboangiitis obliterans [Buerger]

#### I73.8 - Other specified peripheral vascular diseases

#### I73.9 - Peripheral vascular disease, unspecified

#### I79.0 - Disorders of arteries, arterioles and capillaries in diseases classified elsewhere

#### K55.1 - Chronic vascular disorders of intestine

#### K55.8 - Other vascular disorders of intestine

#### K55.9 - Vascular disorder of intestine, unspecified

#### Z95.8 - Presence of other cardiac and vascular implants and grafts

#### Z95.9 - Presence of cardiac and vascular implant and graft, unspecified

### 7 - HYPERTENSION

#### I10.x - Essential (primary) hypertension

#### I11.x - Hypertensive heart disease

#### I12.x - Hypertensive renal disease

#### I13.x - Hypertensive heart and renal disease

#### I15.x - Secondary hypertension

# B - NEUROLOGIC HISTORY:

### 8 - PSYCHOSES

#### F20.x - Schizophrenia

#### F22.x - Persistent delusional disorders

#### F23.x - Acute and transient psychotic disorders

#### F24.x - Induced delusional disorder

#### F25.x - Schizoaffective disorders

#### F28.x - Other nonorganic psychotic disorders

#### F29.x - Unspecified nonorganic psychosis

#### F30.2 - Mania with psychotic symptoms

#### F31.2 - Bipolar affective disorder, current episode manic with psychotic symptoms

#### F31.5 - Bipolar affective disorder, current episode severe depression with psychotic symptoms

### 9 - DEPRESSION

#### F20.4 - Post-schizophrenic depression

#### F31.3 - Bipolar affective disorder, current episode mild or moderate depression

#### F31.4 - Bipolar affective disorder, current episode severe depression without psychotic symptoms

#### F31.5 - Bipolar affective disorder, current episode severe depression with psychotic symptoms

#### F32.x - Depressive episode

#### F33.x - Recurrent depressive disorder

#### F34.1 - Dysthymia

#### F41.2 - Mixed anxiety and depressive disorder

#### F43.2 - Adjustment disorders

### 10 - DEMENTIA

#### F00.x - Dementia in Alzheimer disease

#### F01.x - Vascular dementia

#### F02.x - Dementia in other diseases classified elsewhere

#### F03.x - Unspecified dementia

#### G30.x - Alzheimer disease with early onset

#### G31.x - Other degenerative diseases of nervous system, not elsewhere classified

### 11 - CEREBROVASCULAR DISEASE

#### G45.x - Transient cerebral ischaemic attacks and related syndromes

#### G46.x - Vascular syndromes of brain in cerebrovascular diseases

#### I69.x - Sequelae of cerebrovascular disease

### 12 – HEMIPLEGIA, PARAPLEGIA OR PARALYTIC SYNDROME

#### G04.1 - Tropical spastic paraplegia

#### G11.4 - Hereditary spastic paraplegia

#### G80.1 - Spastic diplegic cerebral palsy

#### G80.2 - Spastic hemiplegic cerebral palsy

#### G81.x - Hemiplegia

#### G82.x - Paraplegia and tetraplegia

#### G83.0 - Diplegia of upper limbs

#### G83.1 - Monoplegia of lower limb

#### G83.2 - Monoplegia of upper limb

#### G83.3 - Monoplegia, unspecified

#### G83.4 - Cauda equina syndrome

#### G83.9 - Paralytic syndrome, unspecified

# C - RESPIRATORY HISTORY

### 13 - CHRONIC OBSTRUCTIVE PULMONARY DISEASE

#### J43.x - Emphysema

#### J44.x - Other chronic obstructive pulmonary disease

### 14 - PULMONARY CIRCULATION DISORDERS

#### I27.x - Primary pulmonary hypertension

#### I28.8 - Other specified diseases of pulmonary vessels

#### I28.9 - Disease of pulmonary vessels, unspecified

### 15 - CHRONIC RESPIRATORY FAILURE

#### J96.1 - Chronic respiratory failure

# D - OTHERS

### 16 – CHRONIC ALCOHOL ABUSES

#### F10 - Mental and behavioural disorders due to use of alcohol

#### E52 - Niacin deficiency [pellagra]

#### G62.1 - Alcoholic polyneuropathy

#### I42.6 - Alcoholic cardiomyopathy

#### K29.2 - Alcoholic gastritis

#### K70.x - Alcoholic liver disease

#### T51.x - Toxic effect of alcohol

#### Z50.2 - Alcohol rehabilitation

#### Z71.4 - Alcohol abuse counselling and surveillance

#### Z72.1 - Alcohol use

### 17 – CANCER: ANY MALIGNANCY, INCLUDING LYMPHOMA AND LEUKEMIA.

#### C00.x to C75.x - Malignant neoplasms, stated or presumed to be primary, of specified sites, except of lymphoid, hematopoietic and related tissue

#### C76.x - Malignant neoplasm of other and ill-defined sites

#### C80.x - Malignant neoplasm, without specification of site

#### C81.x to C96.x - Malignant neoplasms, stated or presumed to be primary, of lymphoid, hematopoietic and related tissue

#### C97.x - Malignant neoplasms of independent (primary) multiple sites

#### Z85.x - Personal history of malignant neoplasm

#### Z86.0 - Personal history of other neoplasms

### 18 - DEFICIENCY ANEMIA

#### D50.x - Iron deficiency anemia

#### D51.x - Vitamin B12 deficiency anemia

#### D52.x - Folate deficiency anemia

#### D53.x - Other nutritional anemia.

### 19 - DIABETES

#### E10.0 to E10.9 - Insulin-dependent diabetes mellitus

#### E11.0 to E11.9 - Non-insulin-dependent diabetes mellitus

#### E12.0 to E12.9 - Malnutrition-related diabetes mellitus

#### E13.0 to E13.9 - Other specified diabetes mellitus

#### E14.0 to E14.9 - Unspecified diabetes mellitus

### 20 - DRUG ABUSES

#### F11.x - Mental and behavioural disorders due to use of opioids

#### F12.x - Mental and behavioural disorders due to use of cannabinoids

#### F13.x - Mental and behavioural disorders due to use of sedatives or hypnotics

#### F14.x - Mental and behavioural disorders due to use of cocaine

#### F15.x - Mental and behavioural disorders due to use of other stimulants, including caffeine

#### F16.x - Mental and behavioural disorders due to use of hallucinogens

#### F18.x - Mental and behavioural disorders due to use of volatile solvents

#### F19.x - Mental and behavioural disorders due to multiple drug use and use of other psychoactive substances

#### Z71.5 - Drug abuse counselling and surveillance

#### Z72.2 - Drug use

### 21 - TRANSPLANTED ORGAN

#### Z94.0 - Kidney transplant status

#### Z94.1 - Heart transplant status

#### Z94.2 - Lung transplant status

#### Z94.3 - Heart and lungs transplant status

#### Z94.4 - Liver transplant status

### 22 - LIVER DISEASE

#### B18.x - Chronic viral hepatitis

#### I85.x - Oesophageal varices

#### I86.4 - Gastric varices

#### I98.2 - Esophageal varices without bleeding in diseases classified elsewhere

#### K70.x - Alcoholic liver disease

#### K71.1 - Toxic liver disease with hepatic necrosis

#### K71.3 - Toxic liver disease with chronic persistent hepatitis

#### K71.4 - Toxic liver disease with chronic lobular hepatitis

#### K71.5 - Toxic liver disease with chronic active hepatitis

#### K71.7 - Toxic liver disease with fibrosis and cirrhosis of liver

#### K72.x - Hepatic failure, not elsewhere classified

#### K73.x - Chronic hepatitis, not elsewhere classified

#### K74.x - Fibrosis and cirrhosis of liver

#### K76.x - Other diseases of liver

#### Z94.4 - Liver transplant status

### 23 - METASTATIC SOLID TUMOR

#### C77.x - Secondary and unspecified malignant neoplasm of lymph nodes

#### C78.x - Secondary malignant neoplasm of respiratory and digestive organs

#### C79.x - Secondary malignant neoplasm of other and unspecified sites

### 24 - OBESITY

#### E66.x - Obesity due to excess calories

### 25 - PREADMISSION DIALYSIS

#### Z99.2 - Dependence on renal dialysis

#### Z49.1 - Extracorporeal dialysis

#### Z49.2 - Other dialysis

### 26 - RENAL FAILURE

#### I12.0 - Hypertensive renal disease with renal failure

#### I13.1 - Hypertensive heart and renal disease with (congestive) heart failure

#### N18.1 - Chronic kidney disease, stage 1

#### N18.2 - Chronic kidney disease, stage 2

#### N18.3 - Chronic kidney disease, stage 3

#### N18.4 - Chronic kidney disease, stage 4

#### N18.5 - Chronic kidney disease, stage 5

#### N18.9 - Chronic kidney disease, unspecified

#### N19.x - Unspecified kidney failure
